# Metropolitan Hospital

**Published:** 1903-05-30

**Authors:** 


### METROPOLITAN HOSPITAL.

### Festival Dinner.

A highly successful festival dinner in connection with the Metropolitan Hospital was given in the Whitehall Rooms of the Hôtel Métropole on Wednesday, May 20th. Lord Howard de Walden was in the chair.

In giving the toast of "Prosperity to the Hospital" Lord Howard de Walden said he happened to know something of the neighbourhood in which the hospital was situated; he had seen for himself the state of affairs and the condition of the poor in that district, and anyone who had done that would feel compelled to give what little assistance he could to remedy the unfortunate conditions that existed. Knowing what he did, he fully appreciated the wisdom of the committee who decided to remove the hospital from Bishopsgate to the Kingsland Road. There was no other hospital near, and from a careful inspection of the institution he had formed the opinion that they were doing their work very well. He had been acquainted with one or two hospitals which suffered from favourable circumstances, and he was almost tempted to think that the more favourable the circumstances the worse as a rule was the condition of the hospital. A circumstance which increased the difficulty of making the appeal which they now did on behalf of the Metropolitan Hospital was that certain other hospitals were at the same time making such large demands upon the public purse. He was inclined to believe that there was considerable foundation for the statement that the public estimated the worth of a charity by the amount of its indebtedness. It was an extremely regrettable state of things, for had the committee realised the circumstances earlier they would probably have been able to run heavily into debt by undertaking building operations for which they had no prospect of paying, but which would have been a valuable asset as it would have enabled them to appeal to the public for support on the substantial ground that they were deeply in debt. After referring to the admirable services rendered to the hospital by the late Mr. Fry and Mr. Leopold de Rothschild, he stated it gave him much pleasure to mention the Ladies' Association.

Mr. Leopold de Rothschild, in acknowledging the toast, congratulated the chairman on his successful *début* as a festival chairman. He had made an excellent speech, marked by humour, good taste, and sympathy. He thought it was what in racing parlance would be called a "cert." that Lord Howard de Walden would often in the future be asked to occupy a position similar to that he now filled. The Metropolitan Hospital was fortunate in having secured Lord Howard de Walden to preside at that dinner; but their good fortune did not end there, for they had secured his services as chairman of the hospital. That appointment would not in any way interfere with the duties so ably performed by the chairman of committees, and he trusted they would find his lordship taking a substantial interest in the working of the hospital. Sixteen years ago no beds were provided in the hospital, but at the present time they had 111 beds provided, and that fact alone gave them justification in appealing for further funds to carry on their work of usefulness in a very poor district. Some people were disposed to praise one hospital to the detriment of another, but his own opinion was that in the hospitals generally the poor were better cared for than was the richest man in his own house. There was every inducement to the charitable public to see that the hospitals were not prevented by lack of funds from carrying on their good work.

Dr. Montague Miller, the Mayor of Hackney, gave the toast of "The Committee of Management." He said that the Metropolitan Hospital was regarded as one of the ornaments of the Borough of Hackney, a borough which contained a population of half a million souls and had over 100 miles of streets. The hospital had done wonders for the poor of the borough. The position of the Committee of Management was not an enviable one. They seemed to be always between the devil and the deep sea, and four qualifications were necessary in every member of the committee. Each must have a good head, good shoulders, a good heart, and a good pocket. Looking down the list he thought the whole of the committee possessed in full the qualifications necessary, and in one particular at least he thought the good pocket must have been nearly a bottomless abyss, and he thanked them most cordially for the valuable work they had done and were doing in the large and patriotic borough of Hackney.

Mr. C. J. Thomas, the Chairman of the Hospital, responded. Their ordinary income had been supplemented by King Edward's Fund, and as a result they had been able to increase the number of their beds to over 100. He alluded with satisfaction to the success which had attended the founding of the Provident Department.

The Secretary (Mr. C. H. Byers) announced that the total amount of the lists handed in that evening was £6,006. The Chairman's list, which included £1,000 from himself (the Chairman) and £1,000 from Viscount Portman, reached £3,545.

Lord Sandhurst gave the toast of "The Medical Staff," and said that the public gave money to the hospitals to be spent and not to be hoarded. It was their business to keep their hospitals up to date and thoroughly efficient, and if they did that they would find that the public would always generously support a good institution. It was when a hospital was allowed to get slack and to fall behind that public support broke down, and he failed to see that there was much to complain of that such was the case.

Dr. Hay and Mr. Goodsall responded on behalf of the medical staff, Sir Edmund Hay Currie gave the toast of "The Visitors" which Mr. Rufus Isaacs, K.C., acknowledged, and Lord Hillingdon gave the toast of "The Chairman."

